# Author Correction: Downregulation of rhodopsin is an effective therapeutic strategy in ameliorating peripherin-2-associated inherited retinal disorders

**DOI:** 10.1038/s41467-024-50140-3

**Published:** 2024-07-19

**Authors:** Christian T. Rutan Woods, Mustafa S. Makia, Tylor R. Lewis, Ryan Crane, Stephanie Zeibak, Paul Yu, Mashal Kakakhel, Carson M. Castillo, Vadim Y. Arshavsky, Muna I. Naash, Muayyad R. Al-Ubaidi

**Affiliations:** 1 Department of Biomedical Engineering, University of Houston, Houston, TX 77204 USA; 2 Department of Ophthalmology, Duke University Medical Center, Durham, NC 27710 USA

**Keywords:** Neuroscience, Diseases, Mechanisms of disease

Correction to: *Nature Communications* 10.1038/s41467-024-48846-5, published online 4 June 2024

The original version of this Article incorrectly duplicated the figure panels in Figure 2C. All conclusions and results in the manuscript are based on the correct figure.

The previous version of figure 2 was:
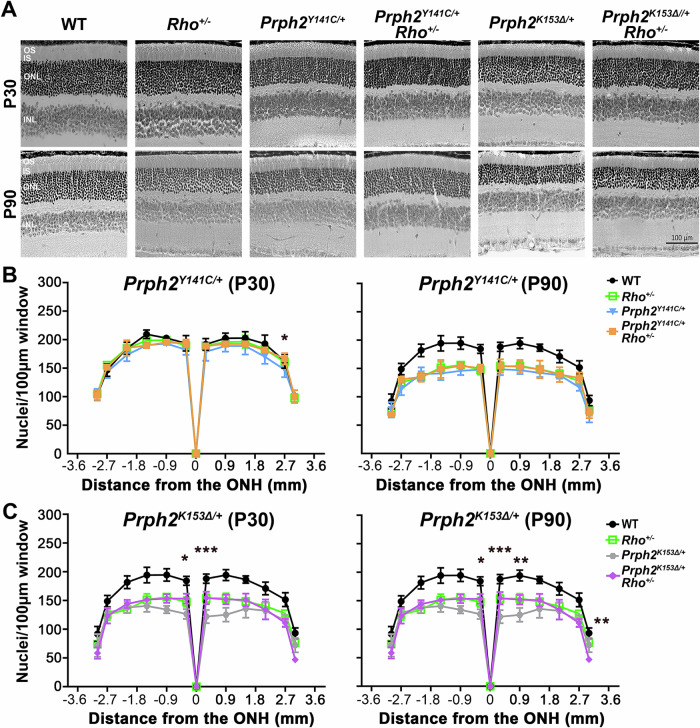


The corrected version is:
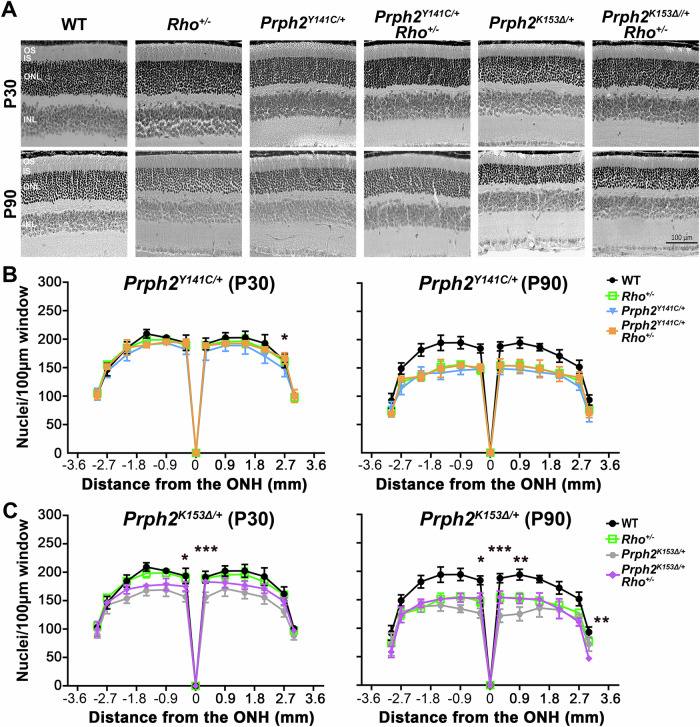


This error has been corrected in both the PDF and HTML versions of the Article.

