# Supplementary material for: Downregulation of rhodopsin is an effective therapeutic strategy in ameliorating peripherin-2-associated inherited retinal disorders
Source: Nat Commun. 2024 Jun 4;15:4756. doi: 10.1038/s41467-024-48846-5 (PMC11150396; doi:10.1038/s41467-024-48846-5)
Supplement: Supplementary file 1 — Supplementary Information [file 41467_2024_48846_MOESM1_ESM.pdf]

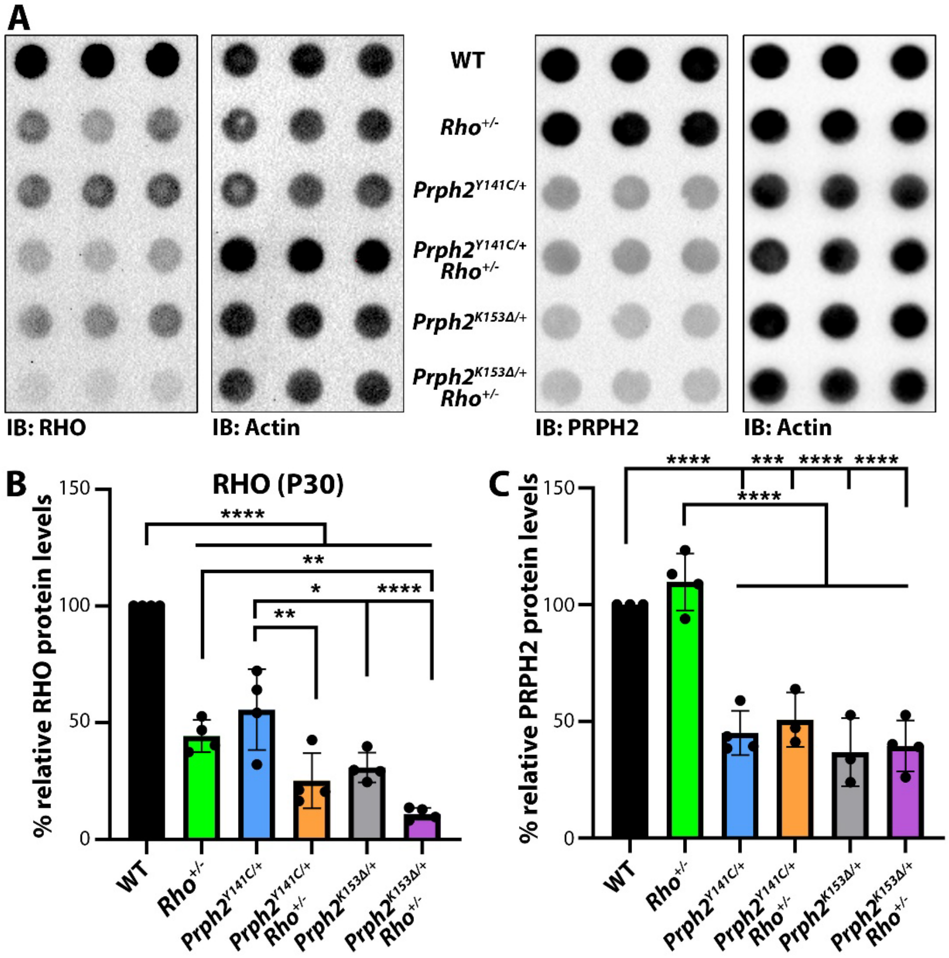

Figure S1: Partial ablation of *Rho* reduces the ratio of RHO to PRPH2 in *Prph2$^{Y141C/+}$* and *Prph2$^{K153\Delta/+}$* retinas. (A) Representative immune-dot blot images of retinal extracts taken at P30 from the listed genotypes. **(B-C)** Signal intensity measurement of the dots in the immunoblots for the listed genotypes at P30 and probed for RHO **(B)** and PRPH2 **(C)** were normalized to actin and plotted relative to WT. Data are presented as mean ± SD. N=3-4 retinas/genotype. $*P<0.05$, $**P<0.01$, $***P<0.001$, $****P<0.001$ by one-way ANOVA ($P<0.0001$ for both RHO and PRPH2) with Tukey's post-hoc comparison.

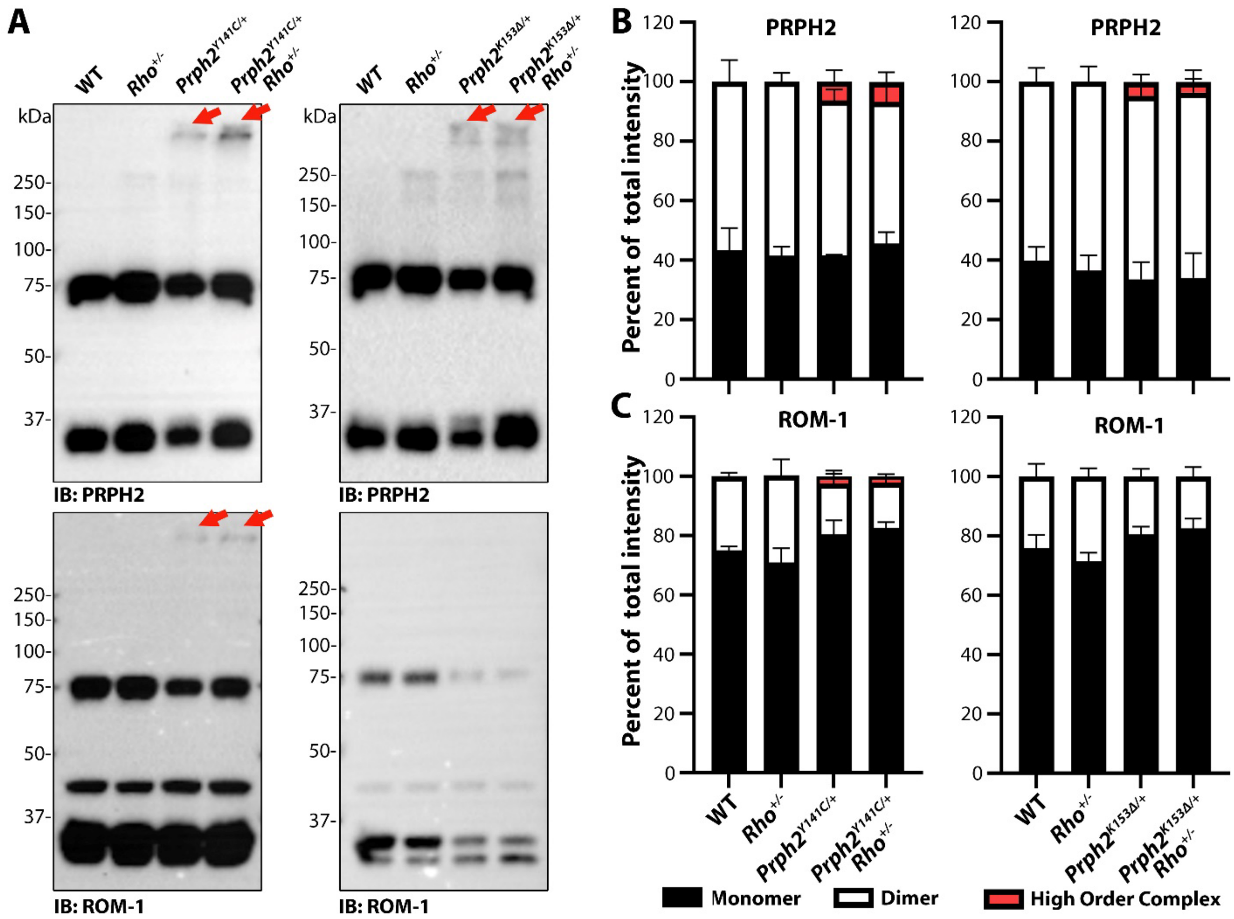

Figure S2: Partial ablation of *Rho* does not affect PRPH2/ROM1 large complex formation. **(A)** Representative immunoblots from P30 retinal extracts from the indicated genotypes and separated on SDS-PAGE, under non-reducing conditions. **(B-C)** Percent of total intensities of monomers, dimers and high order complexes for each genotype were plotted as mean ± SD for PRPH2 and ROM1. Retinal extracts from each model were run with their respective controls to ensure accurate densitometric quantification. N = 3 retinas/genotype. To highlight the differences in high-order complex (indicated by red arrows), representative images have intentionally been saturated. However, all quantification was carried out using unsaturated images.

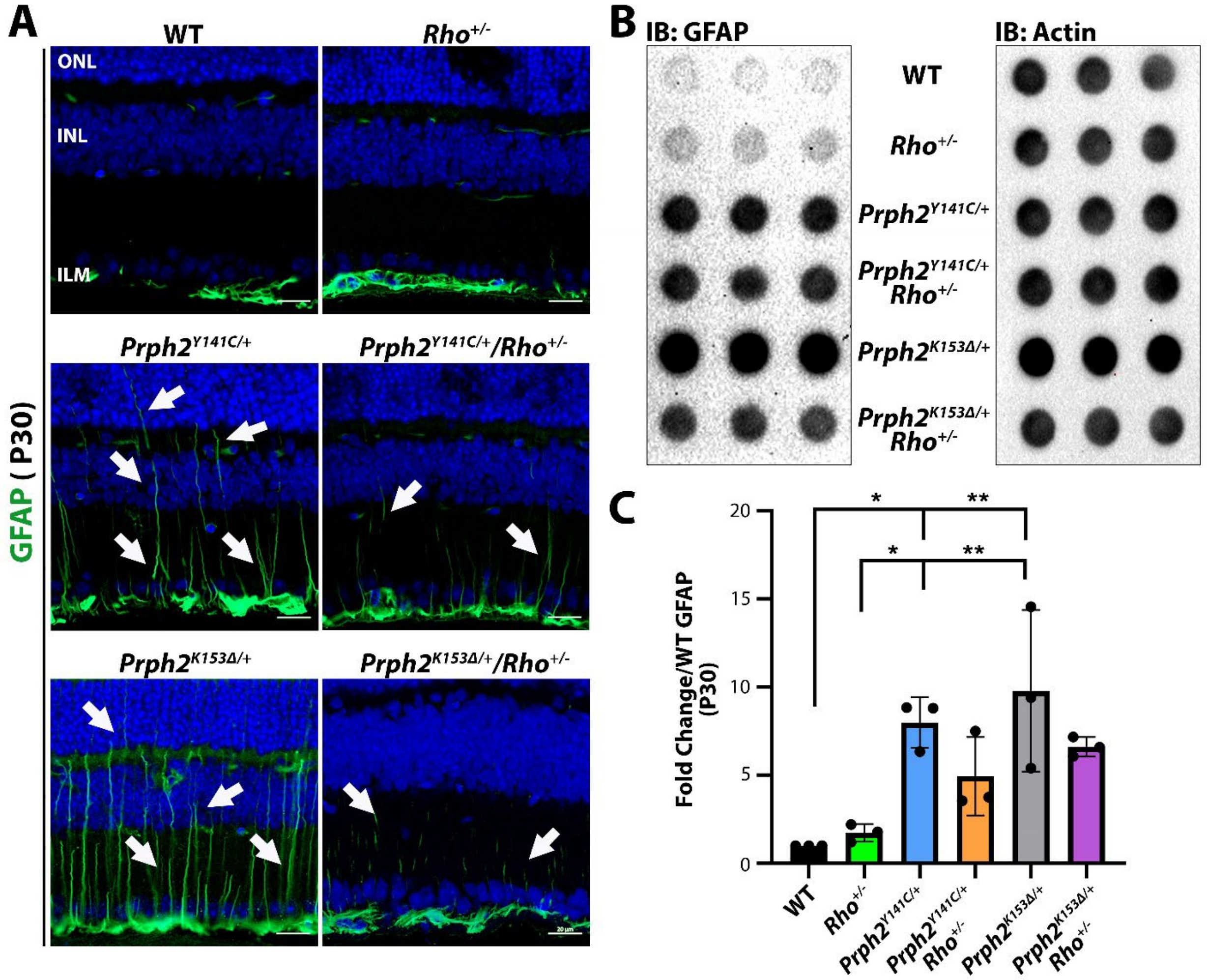

Figure S3: The reduction of RHO reduces gliosis in *Prph2$^{K153\Delta/+}$/Rho$^{+/-}$* and *Prph2$^{Y141C/+}$/Rho$^{+/-}$* retinas. (A) Representative immunostainings of P30 retinal sections from the indicated genotypes and stained for GFAP (green) and DAPI (blue). Arrows are used to mark GFAP infiltration across retinal layers in the models with wild-type RHO levels and their retractions on the *Rho$^{+/-}$* background. **(B)** Representative immunodot blots probed for GFAP (left) and actin (right). **(C)** Fold changes in GFAP relative to WT were quantified from the immunodot blots presented in B and plotted as mean ± SD. Samples from each genetic background were run with their own set of controls to allow for proper densitometric quantification. N= 3-4 replicates/genotype. ∗∗P<0.01, ∗∗∗P<0.001, ∗∗∗∗P<0.001 by one-way ANOVA (P=0.002) with Tukey's post-hoc comparison. Scale bar represents 20 μm.

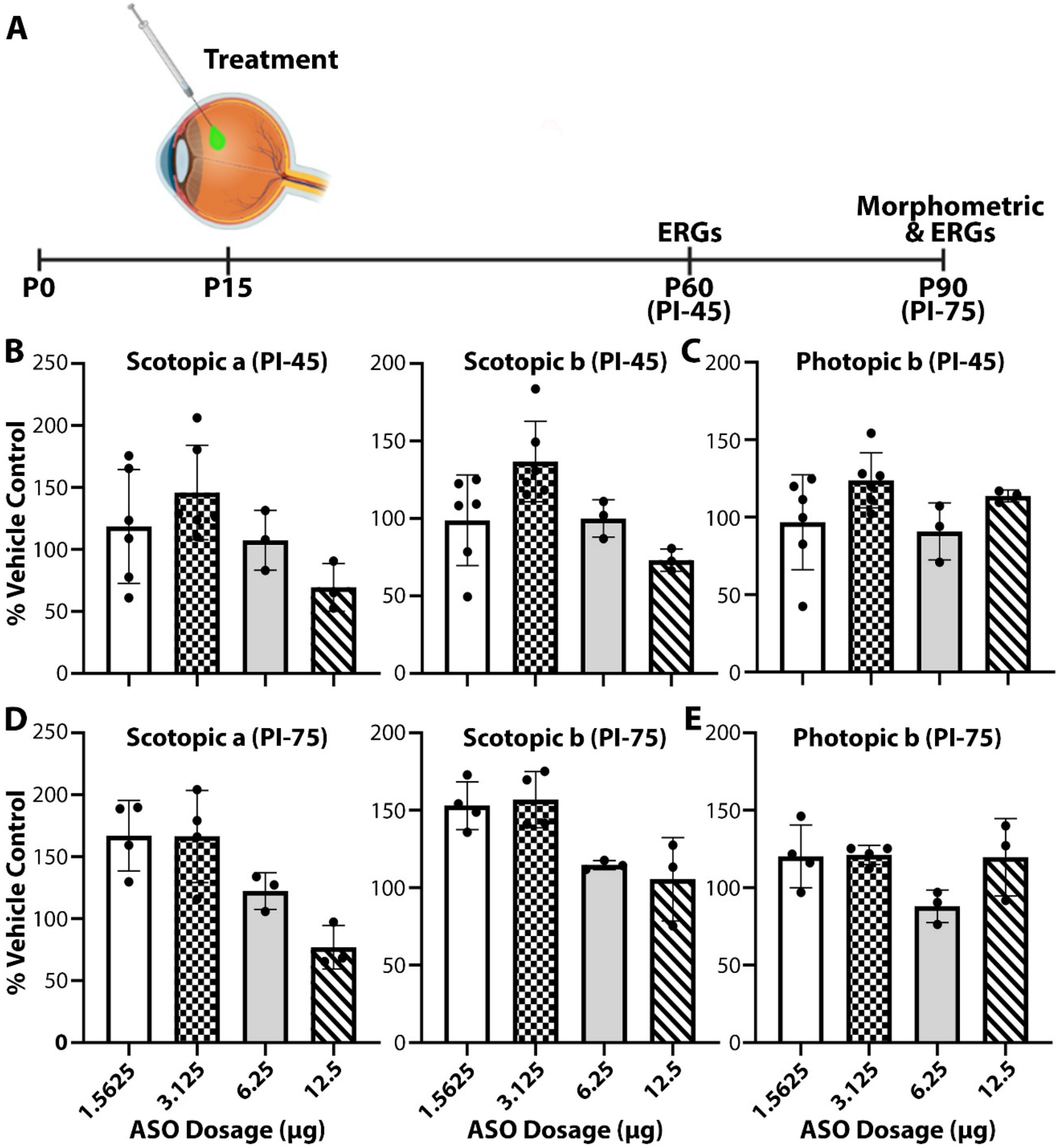

Figure S4: Optimal *mRho* ASO1 dosage for early-stage therapeutic intervention. (A) Design of the titration studies for the early-stage preclinical *mRho* ASO1 dosage created with BioRender.com, released under a Creative Commons Attribution-Non-commercial-NoDervis 4.0 International license. **(B-C)** Scotopic a-, scotopic b-, and photopic b-wave amplitudes are plotted as mean ± SD of the percent of the independent vehicle control for early intervention dosage titrations measured at P60. **(D-E)** Scotopic a-, scotopic b-, and photopic b-wave amplitudes are plotted as mean ± SD of the percent of the independent vehicle control eye for early intervention dosage titrations measured at P90. N=3-6 eyes/treatment condition.

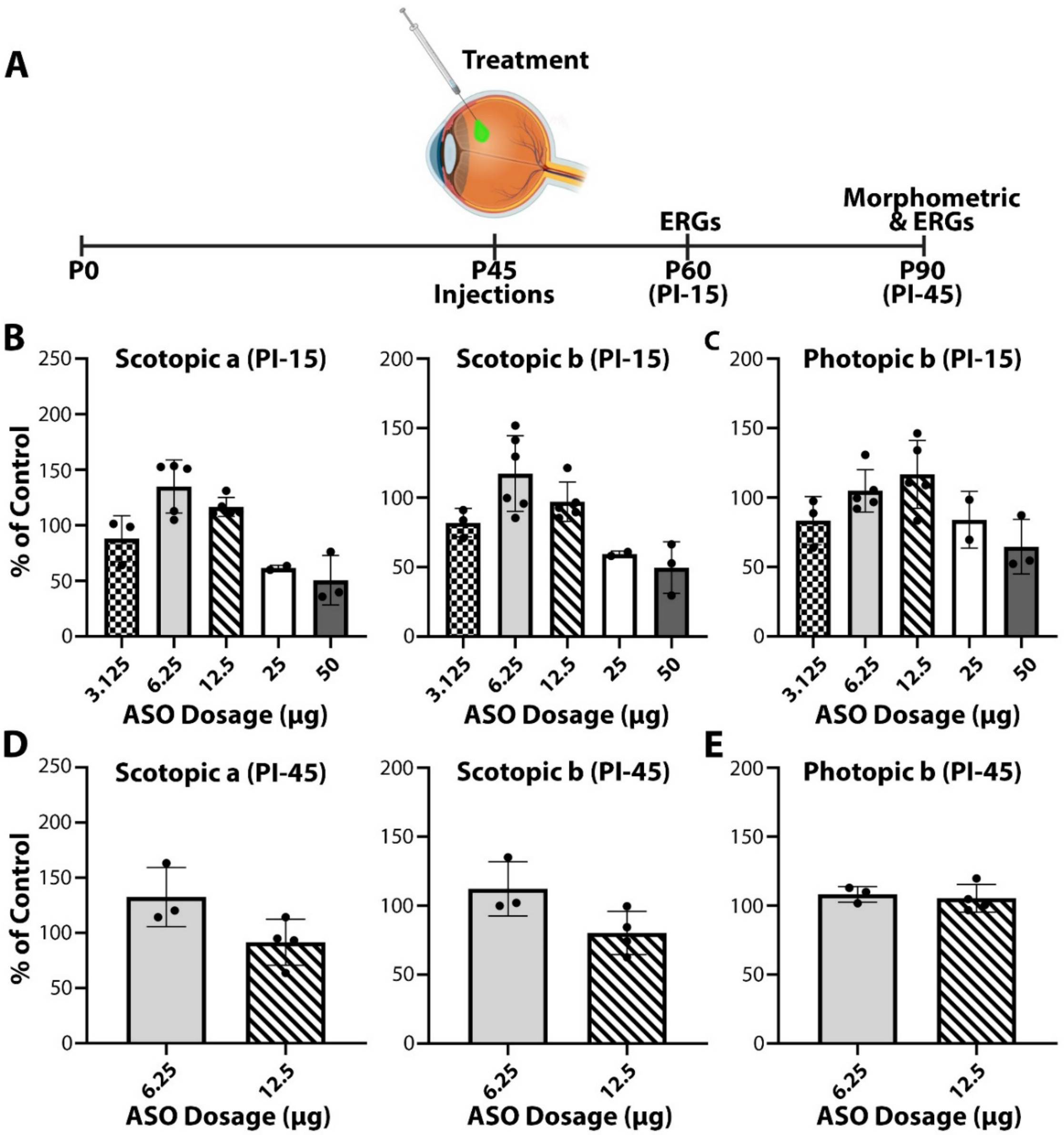

Figure S5: Optimal *mRho* ASO1 dosage for late-stage intervention. (A) Design of preclinical late-stage dosage titration studies created with BioRender.com, released under a Creative Commons Attribution-Non-commercial-NoDervis 4.0 International license. **(B-C)** Scotopic a-, scotopic b-, and photopic b-wave amplitudes are plotted as mean ± SD of the percentage of the contralateral vehicle control for late intervention dosage titrations measured at P60. **(D-E)** Scotopic a-, scotopic b-, and photopic b-wave amplitudes are plotted as mean ± SD of the percentage of the contralateral vehicle control eye for late intervention dosage titrations measured at P90. N=3-6 eyes/treatment condition.

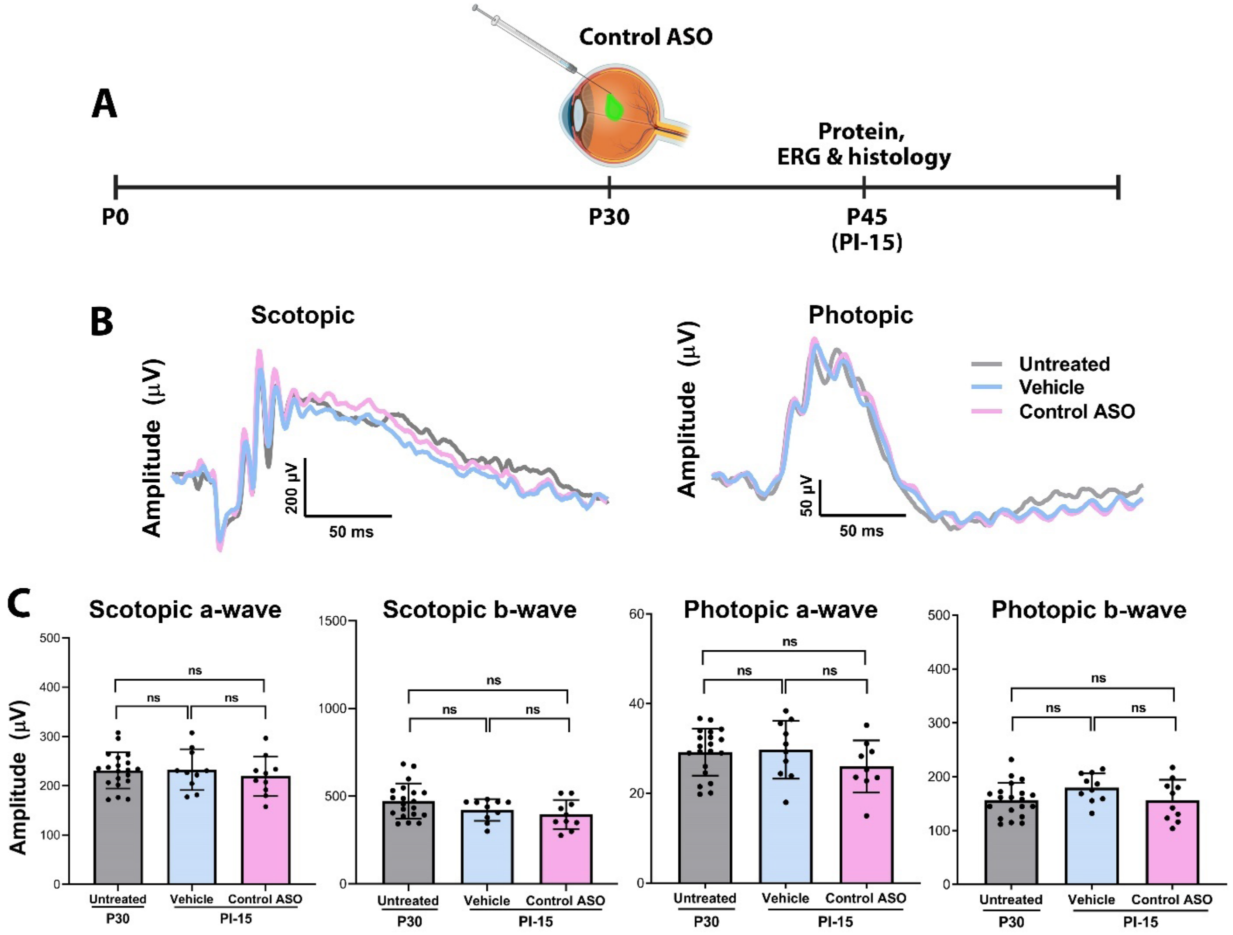

Figure S6: Control ASO does not alter the ERG responses of injected eyes. (A) Schematic representation of the design for injection of the control ASO (6.25 µg) and the functional assessments created with BioRender.com, released under a Creative Commons Attribution-Non-commercial-NoDervis 4.0 International license. **(B)** Representative waveforms of scotopic and photopic responses recorded 15 days post-injection of the control ASO. **(C)** Mean ± SD maximum amplitudes of scotopic a- and b-waves and photopic a- and b-waves of control ASO treated eyes relative to either untreated or vehicle treated eyes. ns: non-significant by one-way ANOVA test (N=20 for uninjected; 9-10 for vehicle or control ASO injected).

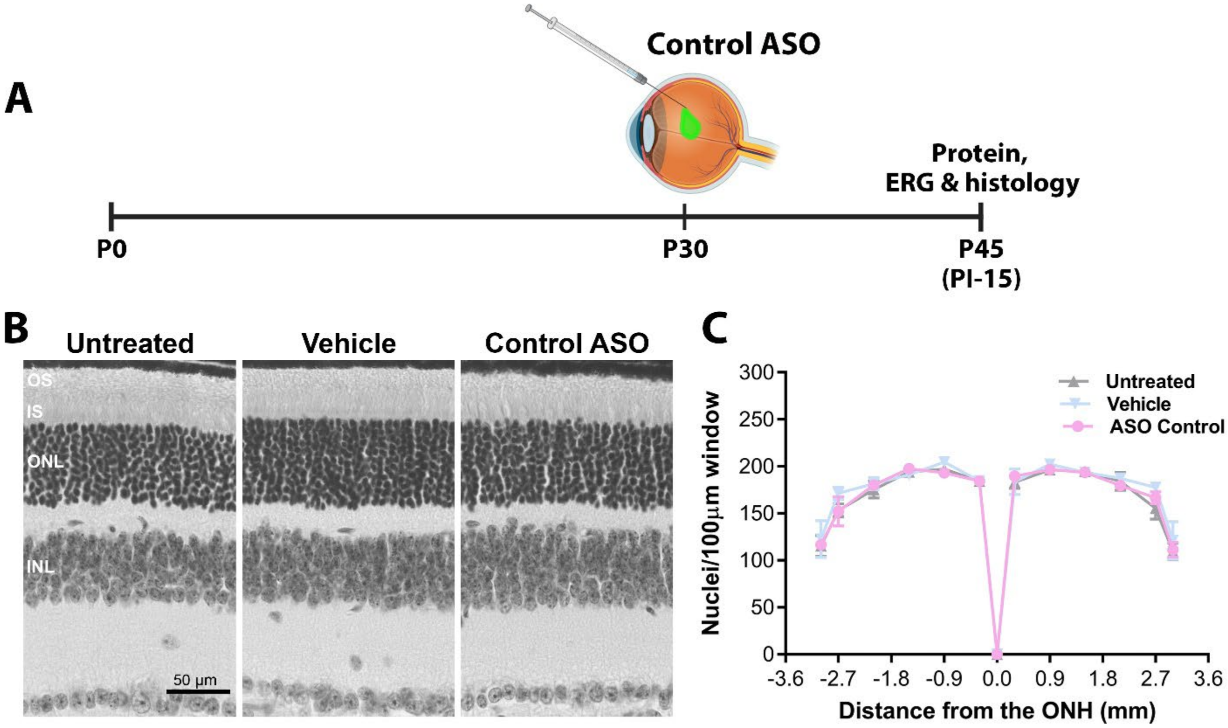

Figure S7: Control ASO does not alter the histologic appearance of the injected eyes. (A) Schematic representation of the design for injection of the control ASO (6.25 µg) and the histologic evaluations created with BioRender.com, released under a Creative Commons Attribution-Non-commercial-NoDervis 4.0 International license. **(B)** Representative light images of retinal cross sections from eyes injected with control ASO, compared to either untreated or vehicle-injected eyes, 15 days post-injection. **(C)** Spidergram representing the count of photoreceptor nuclei in the outer nuclear layer of eyes injected with control ASO, compared to vehicle-injected and untreated eyes. (N=3). Statistical analysis by one-way ANOVA showed lack of significant differences between the groups.

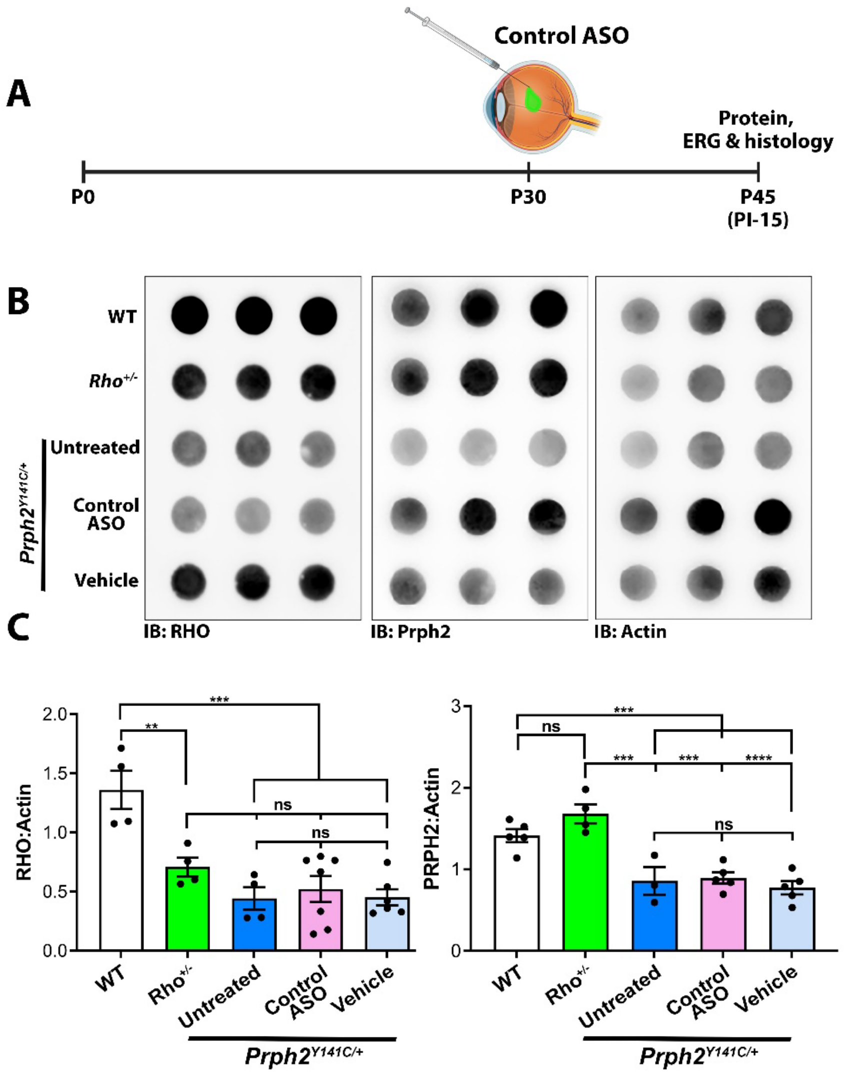

Figure S8: Control ASO does not alter the levels of RHO or PRPH2. (A) Schematic representation of the design for injection of 6.25 µg of the control ASO and the immunoblot analyses created with BioRender.com, released under a Creative Commons Attribution-Non-commercial-NoDervis 4.0 International license. **(B)** Representative immunodot blots were used to assess the levels of RHO and PRPH2 in retinal extracts from eyes injected with control ASO, in comparison to untreated or vehicle-injected eyes, 15 days post-injection. Representative of three independent samples for each treatment are shown. **(C)** Quantification of the levels of RHO (left panel) or PRPH2 (right panel) in retinal extracts from eyes injected with control ASO, in comparison to vehicle-injected or untreated eyes. (N=3 to 7) plotted as mean ± SD. ns, non-significant; $*P<0.05$; $***P<0.001$ by one-way ANOVA.

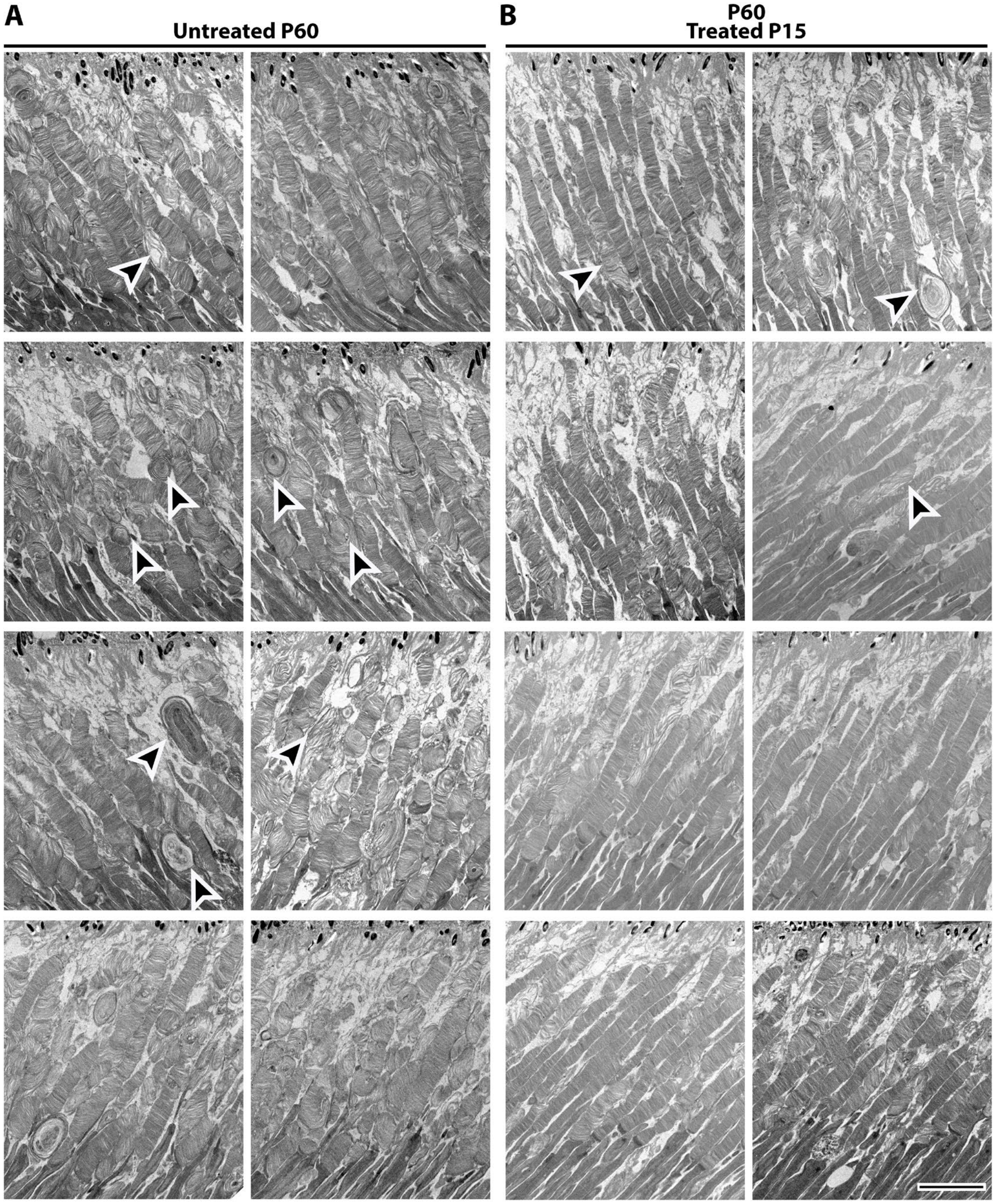

Figure S9: *mRho* ASO1 intervention at P15 leads to enhanced OS ultrastructure and reduced formation of whorl like structures. Representative TEM images captured from retinas at P60 showing untreated contralateral control **(A)** and 3.125 µg *mRho* ASO1 injected eyes at P15 and evaluated at P60 (PI-45) **(B)**. Scale bar, 6 µm. Images are from one animal to illustrate improvements observed throughout the retina. Arrowheads point to irregularities in outer segment discs.

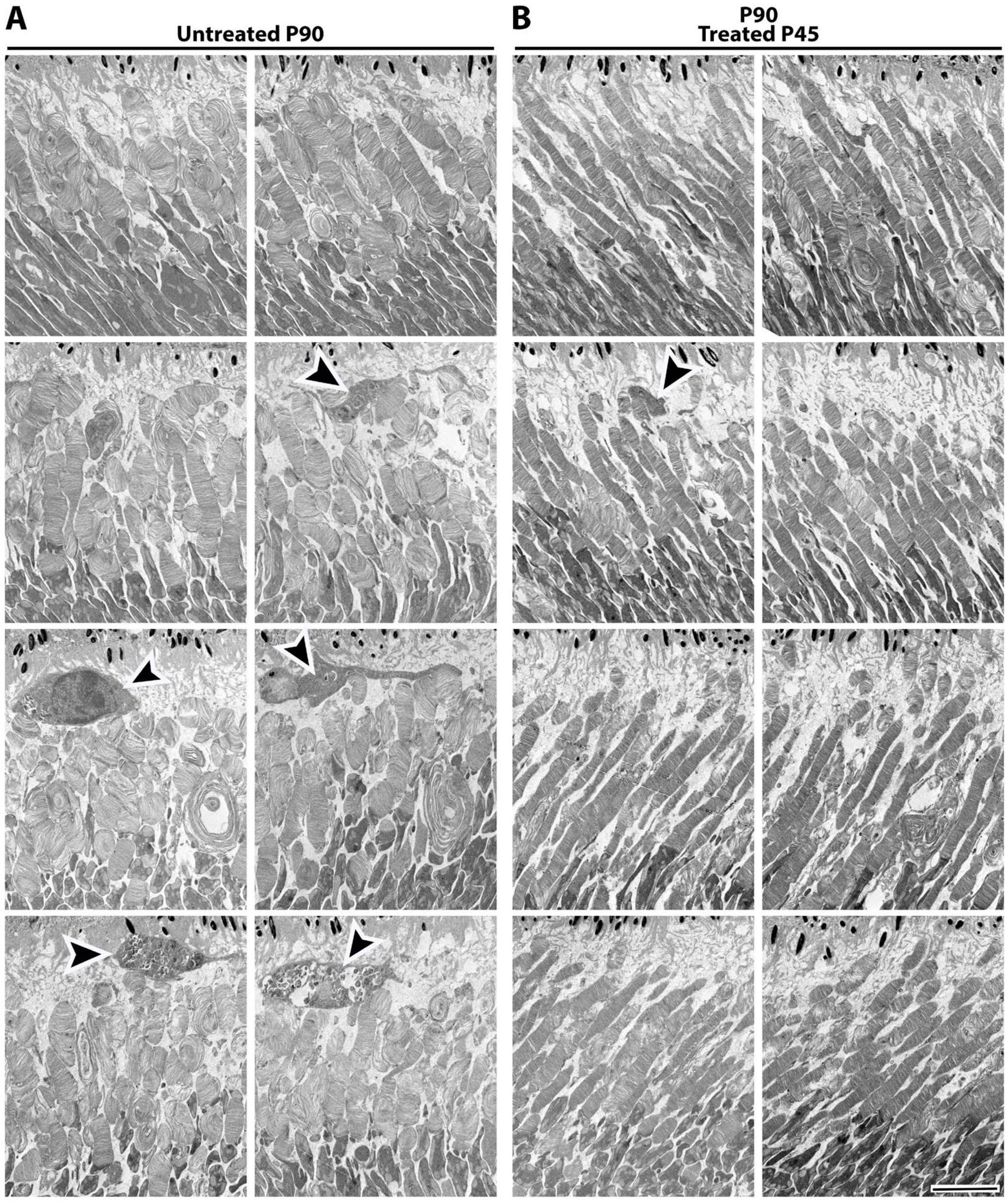

Figure S10: *mRho* ASO1 intervention at P45 leads to enhanced OS ultrastructure, reduced formation of whorl like structures, and decreased infiltration of mononuclear cells. Representative TEM images of retinas at P90 showing untreated contralateral eyes **(A)** and 6.25 µg *mRho* ASO1 treated eyes at P45 and evaluated at P90 (PI-45) **(B)**. Scale bar, 6 µm. Images are from one animal to illustrate the widespread improvements observed throughout the retina. Arrowheads indicate the observed mononuclear cell infiltration in the subretinal space. Number of independent samples is 2.

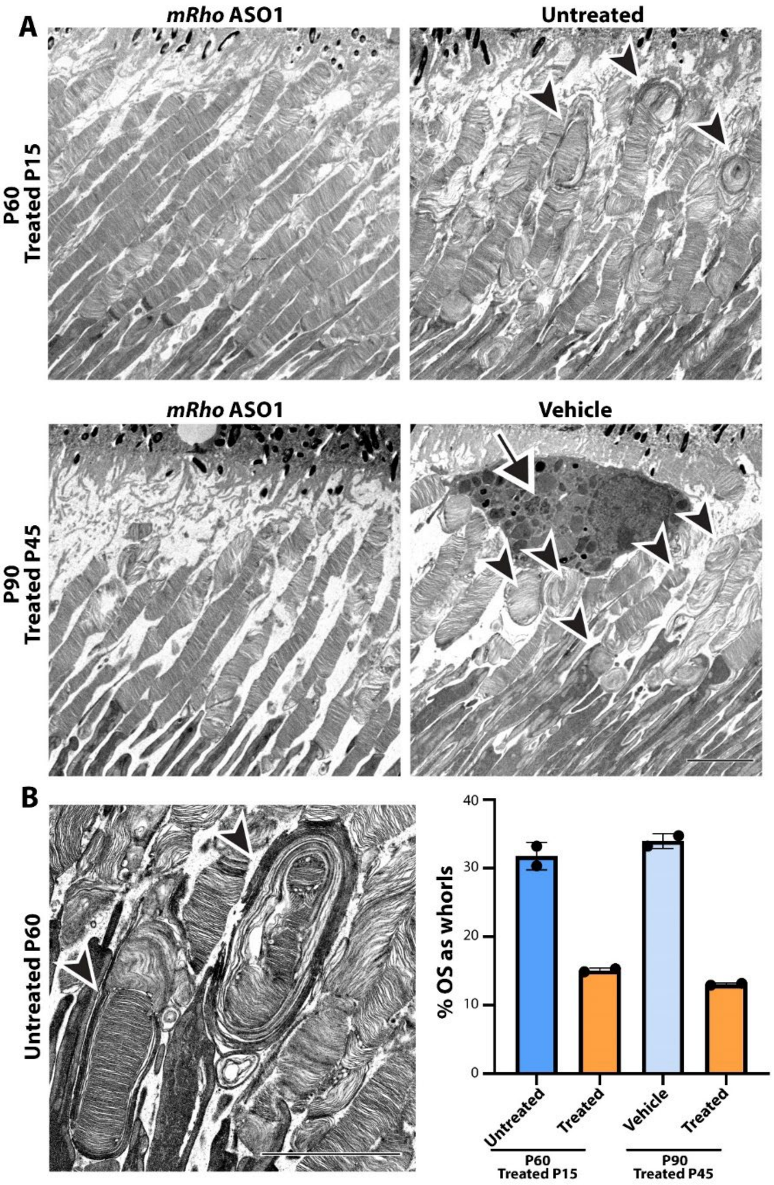

Figure S11: *mRho* ASO1 treatment leads to improvements in ROS ultrastructure and reduced immune cell infiltration. (A) Representative low-magnification TEM images of tannic acid/uranyl acetate-stained retinas from *mRho* ASO1 treated eyes 45 days following treatment at P15 (3.125 µg *mRho* ASO1) and P45 (6.25 µg *mRho* ASO1) and untreated contralateral control eyes. **(B)** A representative image of a whorl-like structure present in P60 uninjected contralateral *Prph2*$^{Y141C/+}$ eye (left) and quantification of whorls presented as a percentage of the total number of counted OSs. N=144-307 OSs counted per retina (right). Number of independent samples is 2 retinas per treatment condition. An arrow indicates mononuclear cell infiltration while arrowheads highlight whorl-like structures. Scale bar, 5 µm. Error bars represent mean ± SD.

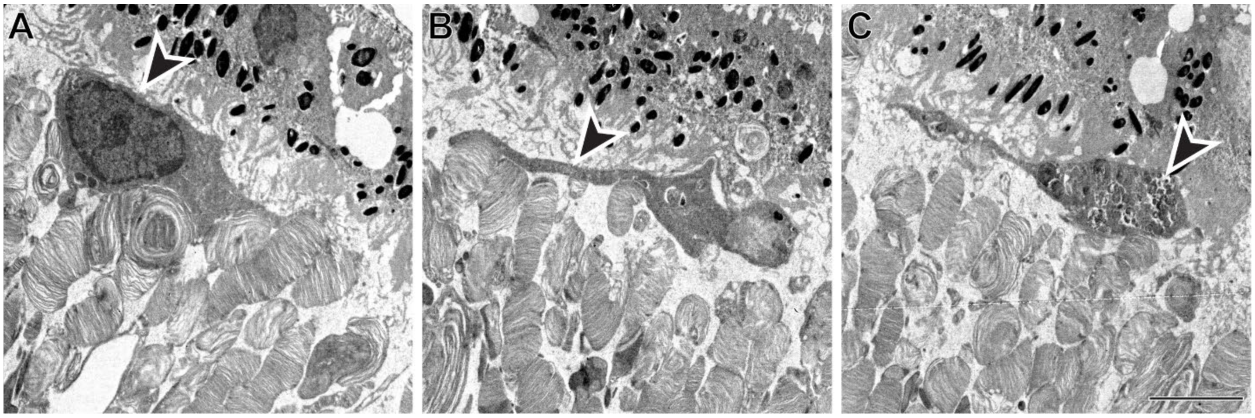

Figure S12: Defining characteristics of presumed microglial immune cell infiltration. Representative TEM images of tannic acid/uranyl acetate-stained retinas from *Prph2*$^{Y141C/+}$ eyes with control injection of PBS at P45 and collected at P90. **(A)** Mononucleated cell located in the subretinal space **(B)**. Extended processes observed as a characteristic of these cells **(C)**. Cells found to possess a large amount of phagocytosed material. Arrowheads highlight: **(A)** nuclei, **(B)** extended processes, and **(C)** phagocytosed material. Scale bar, 5 µm. Number of independent samples is 2.

Supplementary Tables:

Supplementary Table 1: Primers used for qRT-PCR

qRT-PCR Primers		
**Gene of Interest**	**Sequence (5'-3')**	**FWD/REV**
Rho	CACTCCATGGCTACTTCGTCTTT	FWD
	TGGCCCAAATGTTGCTGGATAGTTTTT	REV
Prph2	GGAGGTCAAAGATCGCATCA	FWD
	GCTCCTCAGTCTGATGGTCATA	REV
Gapdh	GAAGGTCGGTGTGAACGG	FWD
	ATGAAGGGGTCGTTGATGGC	REV

Supplementary Table 2: Antibodies used for immunoassays

Antigen	Species	Clone	Application/Concentration	Source
**PRPH2**	Mouse	2B7	1:1,000 (IB)	Millipore Cat# MABN2395
**ROM1**	Mouse	2H5	1:1,000 (IB)	Millipore Cat# MABN1757
**RHO**	Mouse	1D4	1:2,000 (IB)	Santa Cruz Biotechnology Cat# sc-57432
**GFAP**	Mouse	GA5	1:1,000 (IB) 1:500 (IF)	Sigma-Aldrich MAB360
**Actin-HRP**	Mouse	AC-15	1:15,000 (IB)	Sigma-Aldrich Cat# A3854, RRID:AB_262011
**Mouse IgG HRP**	Goat		1:15,000 (IB)	Millipore Cat# AP130P, RRID:AB_91266

Supplementary Table 3: Corresponding one-way ANOVA p-values for Figure 1: Improved rod and cone functions in *Prph2*$^{Y141C/+}$ and *Prph2*$^{K153\Delta/+}$ mice following partial ablation of *Rho*. ∗P<0.05, ∗∗P<0.01, ∗∗∗P<0.001, ∗∗∗∗P<0.0001.

Figure 1 One-way ANOVA of photoreceptor functional responses P-values					
**Model**	**Age**	**Measurement**	**P-value**	**Significance**	**Corresponding Figure**
Prph2$^{Y141C/+}$	P17	Scotopic a-wave	<0.0001	****	Fig. 1C
		Scotopic b-wave	0.002	**	
		Photopic b-wave	0.0371	*	Fig. 1D
	P30	Scotopic a-wave	<0.0001	****	Fig. 1C
		Scotopic b-wave	<0.0001	****	
		Photopic b-wave	0.0212	*	Fig. 1D
	P90	Scotopic a-wave	<0.0001	****	Fig. 1C
		Scotopic b-wave	0.0031	**	
		Photopic b-wave	0.0015	**	Fig. 1D
Prph2$^{K153\Delta/+}$	P17	Scotopic a-wave	<0.0001	****	Fig. 1E
		Scotopic b-wave	<0.0001	****	
		Photopic b-wave	0.0015	**	Fig. 1F
	P30	Scotopic a-wave	<0.0001	****	Fig. 1E
		Scotopic b-wave	<0.0001	****	
		Photopic b-wave	<0.0001	****	Fig. 1F
	P90	Scotopic a-wave	<0.0001	****	Fig. 1E
		Scotopic b-wave	<0.0001	****	
		Photopic b-wave	<0.0001	****	Fig. 1F

Supplementary Table 4: *Prph2$^{Y141C/+}$* expanded morphometric analysis results.

Sample Comparisons	P30 Analysis		P90 Analysis	
**-3.0 mm from Optic Nerve Head**	**Significance**	**P-value**	**Significance**	**P-value**
Prph2$^{+/+}$ vs. *Rho$^{+/-}$*	ns	0.9955	ns	0.0592
Prph2$^{+/+}$ vs. *Prph2$^{Y141C/+}$*	ns	>0.9999	ns	0.109
Prph2$^{+/+}$ vs. *Prph2$^{Y141C/+}$/Rho$^{+/-}$*	ns	0.999	**	0.0068
Rho$^{+/-}$ vs. *Prph2$^{Y141C/+}$*	ns	0.9937	ns	0.9735
Rho$^{+/-}$ vs. *Prph2$^{Y141C/+}$/Rho$^{+/-}$*	ns	0.9821	ns	0.8919
Prph2$^{Y141C/+}$ vs. *Prph2$^{Y141C/+}$/Rho$^{+/-}$*	ns	0.9991	ns	0.6339
**-2.7 mm from Optic Nerve Head**	**Significance**	**P-value**	**Significance**	**P-value**
Prph2$^{+/+}$ vs. *Rho$^{+/-}$*	ns	0.6194	**	0.0059
Prph2$^{+/+}$ vs. *Prph2$^{Y141C/+}$*	ns	0.9562	****	<0.0001
Prph2$^{+/+}$ vs. *Prph2$^{Y141C/+}$/Rho$^{+/-}$*	ns	0.8968	ns	0.0525
Rho$^{+/-}$ vs. *Prph2$^{Y141C/+}$*	ns	0.2743	ns	0.3519
Rho$^{+/-}$ vs. *Prph2$^{Y141C/+}$/Rho$^{+/-}$*	ns	0.9557	ns	0.8919
Prph2$^{Y141C/+}$ vs. *Prph2$^{Y141C/+}$/Rho$^{+/-}$*	ns	0.5901	ns	0.0789
**-2.1 from Optic Nerve Head**	**Significance**	**P-value**	**Significance**	**P-value**
Prph2$^{+/+}$ vs. *Rho$^{+/-}$*	ns	>0.9999	****	<0.0001
Prph2$^{+/+}$ vs. *Prph2$^{Y141C/+}$*	ns	0.3045	****	<0.0001
Prph2$^{+/+}$ vs. *Prph2$^{Y141C/+}$/Rho$^{+/-}$*	ns	0.9997	****	<0.0001
Rho$^{+/-}$ vs. *Prph2$^{Y141C/+}$*	ns	0.3045	ns	0.8714
Rho$^{+/-}$ vs. *Prph2$^{Y141C/+}$/Rho$^{+/-}$*	ns	0.9997	ns	>0.9999
Prph2$^{Y141C/+}$ vs. *Prph2$^{Y141C/+}$/Rho$^{+/-}$*	ns	0.2571	ns	0.8913
**-1.5 mm from Optic Nerve Head**	**Significance**	**P-value**	**Significance**	**P-value**
Prph2$^{+/+}$ vs. *Rho$^{+/-}$*	ns	0.4168	****	<0.0001
Prph2$^{+/+}$ vs. *Prph2$^{Y141C/+}$*	*	0.0232	****	<0.0001
Prph2$^{+/+}$ vs. *Prph2$^{Y141C/+}$/Rho$^{+/-}$*	*	0.034	****	<0.0001

Rho$^{+/-}$ vs. *Prph2*$^{Y141C/+}$	ns	0.598	ns	0.4548
Rho$^{+/-}$ vs. *Prph2*$^{Y141C/+}$/*Rho*$^{+/-}$	ns	0.6194	ns	0.9894
Prph2$^{Y141C/+}$ vs. *Prph2*$^{Y141C/+}$/*Rho*$^{+/-}$	ns	>0.9999	ns	0.6637
**-0.9 mm from Optic Nerve Head**	**Significance**	**P-value**	**Significance**	**P-value**
Prph2$^{+/+}$ vs. *Rho*$^{+/-}$	ns	0.9557	****	<0.0001
Prph2$^{+/+}$ vs. *Prph2*$^{Y141C/+}$	ns	0.4727	****	<0.0001
Prph2$^{+/+}$ vs. *Prph2*$^{Y141C/+}$/*Rho*$^{+/-}$	ns	0.7062	****	<0.0001
Rho$^{+/-}$ vs. *Prph2*$^{Y141C/+}$	ns	0.8057	ns	0.462
Rho$^{+/-}$ vs. *Prph2*$^{Y141C/+}$/*Rho*$^{+/-}$	ns	0.9436	ns	0.998
Prph2$^{Y141C/+}$ vs. *Prph2*$^{Y141C/+}$/*Rho*$^{+/-}$	ns	0.9906	ns	0.5812
**-0.3 mm from Optic Nerve Head**	**Significance**	**P-value**	**Significance**	**P-value**
Prph2$^{+/+}$ vs. *Rho*$^{+/-}$	ns	0.9436	****	<0.0001
Prph2$^{+/+}$ vs. *Prph2*$^{Y141C/+}$	ns	0.3045	****	<0.0001
Prph2$^{+/+}$ vs. *Prph2*$^{Y141C/+}$/*Rho*$^{+/-}$	ns	>0.9999	****	<0.0001
Rho$^{+/-}$ vs. *Prph2*$^{Y141C/+}$	ns	0.6607	ns	>0.9999
Rho$^{+/-}$ vs. *Prph2*$^{Y141C/+}$/*Rho*$^{+/-}$	ns	0.9557	ns	0.9844
Prph2$^{Y141C/+}$ vs. *Prph2*$^{Y141C/+}$/*Rho*$^{+/-}$	ns	0.33	ns	0.9828
**0.0 mm from Optic Nerve Head**	**Significance**	**P-value**	**Significance**	**P-value**
Prph2$^{+/+}$ vs. *Rho*$^{+/-}$	ns	>0.9999	ns	>0.9999
Prph2$^{+/+}$ vs. *Prph2*$^{Y141C/+}$	ns	>0.9999	ns	>0.9999
Prph2$^{+/+}$ vs. *Prph2*$^{Y141C/+}$/*Rho*$^{+/-}$	ns	>0.9999	ns	>0.9999
Rho$^{+/-}$ vs. *Prph2*$^{Y141C/+}$	ns	>0.9999	ns	>0.9999
Rho$^{+/-}$ vs. *Prph2*$^{Y141C/+}$/*Rho*$^{+/-}$	ns	>0.9999	ns	>0.9999
Prph2$^{Y141C/+}$ vs. *Prph2*$^{Y141C/+}$/*Rho*$^{+/-}$	ns	>0.9999	ns	>0.9999
**0.3 mm from Optic Nerve Head**	**Significance**	**P-value**	**Significance**	**P-value**
Prph2$^{+/+}$ vs. *Rho*$^{+/-}$	ns	0.9879	****	<0.0001

Prph2$^{+/+}$ **vs.** ***Prph2***$^{Y141C/+}$	**ns**	**0.2048**	********	**<0.0001**
Prph2$^{+/+}$ **vs.** ***Prph2***$^{Y141C/+}$***/Rho***$^{+/-}$	**ns**	**0.9749**	********	**<0.0001**
Rho$^{+/-}$ **vs.** ***Prph2***$^{Y141C/+}$	**ns**	**0.3704**	**ns**	**0.8815**
Rho$^{+/-}$ **vs.** ***Prph2***$^{Y141C/+}$***/Rho***$^{+/-}$	**ns**	**0.9997**	**ns**	**0.9997**
Prph2$^{Y141C/+}$ **vs.** ***Prph2***$^{Y141C/+}$***/Rho***$^{+/-}$	**ns**	**0.4276**	**ns**	**0.8385**
**0.9 mm from Optic Nerve Head**	**Significance**	**P-value**	**Significance**	**P-value**
Prph2$^{+/+}$ **vs.** ***Rho***$^{+/-}$	**ns**	**0.7868**	********	**<0.0001**
Prph2$^{+/+}$ **vs.** ***Prph2***$^{Y141C/+}$	**ns**	**0.1817**	********	**<0.0001**
Prph2$^{+/+}$ **vs.** ***Prph2***$^{Y141C/+}$***/Rho***$^{+/-}$	**ns**	**0.5016**	********	**<0.0001**
Rho$^{+/-}$ **vs.** ***Prph2***$^{Y141C/+}$	**ns**	**0.729**	**ns**	**0.7288**
Rho$^{+/-}$ **vs.** ***Prph2***$^{Y141C/+}$***/Rho***$^{+/-}$	**ns**	**0.9662**	**ns**	**0.9997**
Prph2$^{Y141C/+}$ **vs.** ***Prph2***$^{Y141C/+}$***/Rho***$^{+/-}$	**ns**	**0.95**	**ns**	**0.7831**
**1.5 mm from Optic Nerve Head**	**Significance**	**P-value**	**Significance**	**P-value**
Prph2$^{+/+}$ **vs.** ***Rho***$^{+/-}$	**ns**	**0.8116**	********	**<0.0001**
Prph2$^{+/+}$ **vs.** ***Prph2***$^{Y141C/+}$	**ns**	**0.1645**	********	**<0.0001**
Prph2$^{+/+}$ **vs.** ***Prph2***$^{Y141C/+}$***/Rho***$^{+/-}$	**ns**	**0.4728**	********	**<0.0001**
Rho$^{+/-}$ **vs.** ***Prph2***$^{Y141C/+}$	**ns**	**0.6685**	**ns**	**0.8023**
Rho$^{+/-}$ **vs.** ***Prph2***$^{Y141C/+}$***/Rho***$^{+/-}$	**ns**	**0.9436**	**ns**	**>0.9999**
Prph2$^{Y141C/+}$ **vs.** ***Prph2***$^{Y141C/+}$***/Rho***$^{+/-}$	**ns**	**0.95**	**ns**	**0.8023**
**2.1 mm from Optic Nerve Head**	**Significance**	**P-value**	**Significance**	**P-value**
Prph2$^{+/+}$ **vs.** ***Rho***$^{+/-}$	**ns**	**0.5308**	*******	**0.0001**
Prph2$^{+/+}$ **vs.** ***Prph2***$^{Y141C/+}$	******	**0.0046**	********	**<0.0001**
Prph2$^{+/+}$ **vs.** ***Prph2***$^{Y141C/+}$***/Rho***$^{+/-}$	**ns**	**0.4168**	********	**<0.0001**
Rho$^{+/-}$ **vs.** ***Prph2***$^{Y141C/+}$	**ns**	**0.2**	**ns**	**0.9872**
Rho$^{+/-}$ **vs.** ***Prph2***$^{Y141C/+}$***/Rho***$^{+/-}$	**ns**	**0.9977**	**ns**	**0.998**
Prph2$^{Y141C/+}$ **vs.** ***Prph2***$^{Y141C/+}$***/Rho***$^{+/-}$	**ns**	**0.2861**	**ns**	**0.999**

**2.7 mm from Optic Nerve Head**	**Significance**	**P-value**	**Significance**	**P-value**
Prph2 $^{+/+}$ **vs.** ***Rho*** $^{+/-}$	**ns**	**0.9977**	******	**0.0041**
Prph2 $^{+/+}$ **vs.** ***Prph2*** $^{Y141C/+}$	**ns**	**0.1171**	********	**<0.0001**
Prph2 $^{+/+}$ **vs.** ***Prph2*** $^{Y141C/+}$ ***/Rho*** $^{+/-}$	**ns**	**0.9297**	**ns**	**0.0509**
Rho $^{+/-}$ **vs.** ***Prph2*** $^{Y141C/+}$	**ns**	**0.1773**	**ns**	**0.4123**
Rho $^{+/-}$ **vs.** ***Prph2*** $^{Y141C/+}$ ***/Rho*** $^{+/-}$	**ns**	**0.8572**	**ns**	**0.8535**
Prph2 $^{Y141C/+}$ **vs.** ***Prph2*** $^{Y141C/+}$ ***/Rho*** $^{+/-}$	*****	**0.0232**	**ns**	**0.0812**
**3.0 mm from Optic Nerve Head**	**Significance**	**P-value**	**Significance**	**P-value**
Prph2 $^{+/+}$ **vs.** ***Rho*** $^{+/-}$	**ns**	**0.9662**	**ns**	**0.0883**
Prph2 $^{+/+}$ **vs.** ***Prph2*** $^{Y141C/+}$	**ns**	**0.9697**	*******	**0.0004**
Prph2 $^{+/+}$ **vs.** ***Prph2*** $^{Y141C/+}$ ***/Rho*** $^{+/-}$	**ns**	**0.999**	*****	**0.0114**
Rho $^{+/-}$ **vs.** ***Prph2*** $^{Y141C/+}$	**ns**	**>0.9999**	**ns**	**0.4913**
Rho $^{+/-}$ **vs.** ***Prph2*** $^{Y141C/+}$ ***/Rho*** $^{+/-}$	**ns**	**0.9879**	**ns**	**0.8919**
Prph2 $^{Y141C/+}$ **vs.** ***Prph2*** $^{Y141C/+}$ ***/Rho*** $^{+/-}$	**ns**	**0.9906**	**ns**	**0.9137**

Supplementary Table 5: $Prph2^{K153\Delta/+}$ expanded morphometric analysis results.

Sample Comparisons	P30 Analysis		P90 Analysis	
**-3.0 mm from Optic Nerve Head**	**Significance**	**P-value**	**Significance**	**P-value**
$Prph2^{+/+}$ vs. $Rho^{+/-}$	ns	0.9955	ns	0.0948
$Prph2^{+/+}$ vs. $Prph2^{K153\Delta/+}$	ns	0.2426	*	0.0262
$Prph2^{+/+}$ vs. $Prph2^{K153\Delta/+}/Rho^{+/-}$	ns	0.9751	***	0.0002
$Rho^{+/-}$ vs. $Prph2^{K153\Delta/+}$	ns	0.1503	ns	0.9909
$Rho^{+/-}$ vs. $Prph2^{K153\Delta/+}/Rho^{+/-}$	ns	0.9147	ns	0.2378
$Prph2^{K153\Delta/+}$ vs. $Prph2^{K153\Delta/+}/Rho^{+/-}$	ns	0.4826	ns	0.3159
**-2.7 mm from Optic Nerve Head**	**Significance**	**P-value**	**Significance**	**P-value**
$Prph2^{+/+}$ vs. $Rho^{+/-}$	ns	0.6213	*	0.0132
$Prph2^{+/+}$ vs. $Prph2^{K153\Delta/+}$	ns	0.912	*	0.0237
$Prph2^{+/+}$ vs. $Prph2^{K153\Delta/+}/Rho^{+/-}$	ns	>0.9999	**	0.0058
$Rho^{+/-}$ vs. $Prph2^{K153\Delta/+}$	ns	0.2116	ns	0.9755
$Rho^{+/-}$ vs. $Prph2^{K153\Delta/+}/Rho^{+/-}$	ns	0.6213	ns	0.9947
$Prph2^{K153\Delta/+}$ vs. $Prph2^{K153\Delta/+}/Rho^{+/-}$	ns	0.912	ns	0.9043
**-2.1 mm from Optic Nerve Head**	**Significance**	**P-value**	**Significance**	**P-value**
$Prph2^{+/+}$ vs. $Rho^{+/-}$	ns	>0.9999	****	<0.0001
$Prph2^{+/+}$ vs. $Prph2^{K153\Delta/+}$	****	<0.0001	****	<0.0001
$Prph2^{+/+}$ vs. $Prph2^{K153\Delta/+}/Rho^{+/-}$	ns	0.1763	****	<0.0001
$Rho^{+/-}$ vs. $Prph2^{K153\Delta/+}$	****	<0.0001	ns	0.9716
$Rho^{+/-}$ vs. $Prph2^{K153\Delta/+}/Rho^{+/-}$	ns	0.1763	ns	0.6594
$Prph2^{K153\Delta/+}$ vs. $Prph2^{K153\Delta/+}/Rho^{+/-}$	ns	0.0776	ns	0.8588
**-1.5 mm from Optic Nerve Head**	**Significance**	**P-value**	**Significance**	**P-value**
$Prph2^{+/+}$ vs. $Rho^{+/-}$	ns	0.4191	****	<0.0001
$Prph2^{+/+}$ vs. $Prph2^{K153\Delta/+}$	****	<0.0001	****	<0.0001
$Prph2^{+/+}$ vs. $Prph2^{K153\Delta/+}/Rho^{+/-}$	****	<0.0001	****	<0.0001

Rho$^{+/-}$ vs. *Prph2*$^{K153\Delta/+}$	****	<0.0001	ns	0.485
Rho$^{+/-}$ vs. *Prph2*$^{K153\Delta/+}$/*Rho*$^{+/-}$	*	0.0138	ns	>0.9999
Prph2$^{K153\Delta/+}$ vs. *Prph2*$^{K153\Delta/+}$/*Rho*$^{+/-}$	ns	0.6	ns	0.4581
**-0.9 mm from Optic Nerve Head**	**Significance**	**P-value**	**Significance**	**P-value**
Prph2$^{+/+}$ vs. *Rho*$^{+/-}$	ns	0.956	****	<0.0001
Prph2$^{+/+}$ vs. *Prph2*$^{K153\Delta/+}$	****	<0.0001	****	<0.0001
Prph2$^{+/+}$ vs. *Prph2*$^{K153\Delta/+}$/*Rho*$^{+/-}$	**	0.0059	****	<0.0001
Rho$^{+/-}$ vs. *Prph2*$^{K153\Delta/+}$	***	0.0001	*	0.0248
Rho$^{+/-}$ vs. *Prph2*$^{K153\Delta/+}$/*Rho*$^{+/-}$	*	0.0269	ns	0.9917
Prph2$^{K153\Delta/+}$ vs. *Prph2*$^{K153\Delta/+}$/*Rho*$^{+/-}$	ns	0.475	ns	0.0561
**-0.3 mm from Optic Nerve Head**	**Significance**	**P-value**	**Significance**	**P-value**
Prph2$^{+/+}$ vs. *Rho*$^{+/-}$	ns	0.944	****	<0.0001
Prph2$^{+/+}$ vs. *Prph2*$^{K153\Delta/+}$	****	<0.0001	****	<0.0001
Prph2$^{+/+}$ vs. *Prph2*$^{K153\Delta/+}$/*Rho*$^{+/-}$	*	0.0498	***	0.0005
Rho$^{+/-}$ vs. *Prph2*$^{K153\Delta/+}$	****	<0.0001	*	0.0307
Rho$^{+/-}$ vs. *Prph2*$^{K153\Delta/+}$/*Rho*$^{+/-}$	ns	0.1763	ns	0.8976
Prph2$^{K153\Delta/+}$ vs. *Prph2*$^{K153\Delta/+}$/*Rho*$^{+/-}$	*	0.0367	**	0.0031
**0.0 mm from Optic Nerve Head**	**Significance**	**P-value**	**Significance**	**P-value**
Prph2$^{+/+}$ vs. *Rho*$^{+/-}$	ns	>0.9999	ns	>0.9999
Prph2$^{+/+}$ vs. *Prph2*$^{K153\Delta/+}$	ns	>0.9999	ns	>0.9999
Prph2$^{+/+}$ vs. *Prph2*$^{K153\Delta/+}$/*Rho*$^{+/-}$	ns	>0.9999	ns	>0.9999
Rho$^{+/-}$ vs. *Prph2*$^{K153\Delta/+}$	ns	>0.9999	ns	>0.9999
Rho$^{+/-}$ vs. *Prph2*$^{K153\Delta/+}$/*Rho*$^{+/-}$	ns	>0.9999	ns	>0.9999
Prph2$^{K153\Delta/+}$ vs. *Prph2*$^{K153\Delta/+}$/*Rho*$^{+/-}$	ns	>0.9999	ns	>0.9999
**0.3 mm from Optic Nerve Head**	**Significance**	**P-value**	**Significance**	**P-value**
Prph2$^{+/+}$ vs. *Rho*$^{+/-}$	ns	0.988	****	<0.0001

Prph2$^{+/+}$ vs. *Prph2*$^{K153\Delta/+}$	****	<0.0001	****	<0.0001
Prph2$^{+/+}$ vs. *Prph2*$^{K153\Delta/+}$/*Rho*$^{+/-}$	ns	0.6506	***	0.0001
Rho$^{+/-}$ vs. *Prph2*$^{K153\Delta/+}$	****	<0.0001	***	0.0003
Rho$^{+/-}$ vs. *Prph2*$^{K153\Delta/+}$/*Rho*$^{+/-}$	ns	0.8362	ns	0.9993
Prph2$^{K153\Delta/+}$ vs. *Prph2*$^{K153\Delta/+}$/*Rho*$^{+/-}$	***	0.0004	***	0.0002
**0.9 mm from Optic Nerve Head**	**Significance**	**P-value**	**Significance**	**P-value**
Prph2$^{+/+}$ vs. *Rho*$^{+/-}$	ns	0.7881	****	<0.0001
Prph2$^{+/+}$ vs. *Prph2*$^{K153\Delta/+}$	****	<0.0001	****	<0.0001
Prph2$^{+/+}$ vs. *Prph2*$^{K153\Delta/+}$/*Rho*$^{+/-}$	*	0.0237	****	<0.0001
Rho$^{+/-}$ vs. *Prph2*$^{K153\Delta/+}$	**	0.0022	**	0.0013
Rho$^{+/-}$ vs. *Prph2*$^{K153\Delta/+}$/*Rho*$^{+/-}$	ns	0.2109	ns	0.9947
Prph2$^{K153\Delta/+}$ vs. *Prph2*$^{K153\Delta/+}$/*Rho*$^{+/-}$	ns	0.408	**	0.0033
**1.5 mm from Optic Nerve Head**	**Significance**	**P-value**	**Significance**	**P-value**
Prph2$^{+/+}$ vs. *Rho*$^{+/-}$	ns	0.8128	****	<0.0001
Prph2$^{+/+}$ vs. *Prph2*$^{K153\Delta/+}$	****	<0.0001	****	<0.0001
Prph2$^{+/+}$ vs. *Prph2*$^{K153\Delta/+}$/*Rho*$^{+/-}$	**	0.0032	****	<0.0001
Rho$^{+/-}$ vs. *Prph2*$^{K153\Delta/+}$	****	<0.0001	ns	0.3505
Rho$^{+/-}$ vs. *Prph2*$^{K153\Delta/+}$/*Rho*$^{+/-}$	*	0.0442	ns	0.9877
Prph2$^{K153\Delta/+}$ vs. *Prph2*$^{K153\Delta/+}$/*Rho*$^{+/-}$	ns	0.1972	ns	0.1901
**2.1 mm from Optic Nerve Head**	**Significance**	**P-value**	**Significance**	**P-value**
Prph2$^{+/+}$ vs. *Rho*$^{+/-}$	ns	0.533	***	0.0005
Prph2$^{+/+}$ vs. *Prph2*$^{K153\Delta/+}$	****	<0.0001	****	<0.0001
Prph2$^{+/+}$ vs. *Prph2*$^{K153\Delta/+}$/*Rho*$^{+/-}$	*	0.0159	****	<0.0001
Rho$^{+/-}$ vs. *Prph2*$^{K153\Delta/+}$	***	0.0006	ns	0.7116
Rho$^{+/-}$ vs. *Prph2*$^{K153\Delta/+}$/*Rho*$^{+/-}$	ns	0.3408	ns	0.8976
Prph2$^{K153\Delta/+}$ vs. *Prph2*$^{K153\Delta/+}$/*Rho*$^{+/-}$	ns	0.1186	ns	0.9888

2.7 mm from Optic Nerve Head	Significance	P-value	Significance	P-value
$Prph2^{+/+}$ vs. $Rho^{+/-}$	ns	0.9977	**	0.0097
$Prph2^{+/+}$ vs. $Prph2^{K153\Delta/+}$	****	<0.0001	****	<0.0001
$Prph2^{+/+}$ vs. $Prph2^{K153\Delta/+}/Rho^{+/-}$	ns	0.271	****	<0.0001
$Rho^{+/-}$ vs. $Prph2^{K153\Delta/+}$	***	0.0001	ns	0.3505
$Rho^{+/-}$ vs. $Prph2^{K153\Delta/+}/Rho^{+/-}$	ns	0.366	ns	0.1888
$Prph2^{K153\Delta/+}$ vs. $Prph2^{K153\Delta/+}/Rho^{+/-}$	*	0.0446	ns	0.9603
**3.0 mm from Optic Nerve Head**	**Significance**	**P-value**	**Significance**	**P-value**
$Prph2^{+/+}$ vs. $Rho^{+/-}$	ns	0.9664	ns	0.1331
$Prph2^{+/+}$ vs. $Prph2^{K153\Delta/+}$	ns	0.2481	*	0.0175
$Prph2^{+/+}$ vs. $Prph2^{K153\Delta/+}/Rho^{+/-}$	ns	0.944	****	<0.0001
$Rho^{+/-}$ vs. $Prph2^{K153\Delta/+}$	ns	0.5213	ns	0.9374
$Rho^{+/-}$ vs. $Prph2^{K153\Delta/+}/Rho^{+/-}$	ns	0.9997	**	0.0021
$Prph2^{K153\Delta/+}$ vs. $Prph2^{K153\Delta/+}/Rho^{+/-}$	ns	0.5842	**	0.0062
