# Supplementary material for: Downregulation of rhodopsin is an effective therapeutic strategy in ameliorating peripherin-2-associated inherited retinal disorders
Source: Nat Commun. 2024 Jun 4;15:4756. doi: 10.1038/s41467-024-48846-5 (PMC11150396; doi:10.1038/s41467-024-48846-5)
Supplement: Supplementary file 3 — Reporting Summary [file 41467_2024_48846_MOESM3_ESM.pdf]

nature portfolio

Corresponding author(s): Muayyad Al-Ubaidi
Last updated by author(s): Feb 26, 2024

Reporting Summary

Nature Portfolio wishes to improve the reproducibility of the work that we publish. This form provides structure for consistency and transparency in reporting. For further information on Nature Portfolio policies, see our Editorial Policies and the Editorial Policy Checklist.

Statistics

For all statistical analyses, confirm that the following items are present in the figure legend, table legend, main text, or Methods section.

n/a	Confirmed	
☐	☒	The exact sample size (*n*) for each experimental group/condition, given as a discrete number and unit of measurement
☐	☒	A statement on whether measurements were taken from distinct samples or whether the same sample was measured repeatedly
☐	☒	The statistical test(s) used AND whether they are one- or two-sided<br>*Only common tests should be described solely by name; describe more complex techniques in the Methods section.*
☒	☐	A description of all covariates tested
☐	☒	A description of any assumptions or corrections, such as tests of normality and adjustment for multiple comparisons
☐	☒	A full description of the statistical parameters including central tendency (e.g. means) or other basic estimates (e.g. regression coefficient) AND variation (e.g. standard deviation) or associated estimates of uncertainty (e.g. confidence intervals)
☐	☒	For null hypothesis testing, the test statistic (e.g. *F*, *t*, *r*) with confidence intervals, effect sizes, degrees of freedom and *P* value noted<br>*Give P values as exact values whenever suitable.*
☒	☐	For Bayesian analysis, information on the choice of priors and Markov chain Monte Carlo settings
☒	☐	For hierarchical and complex designs, identification of the appropriate level for tests and full reporting of outcomes
☒	☐	Estimates of effect sizes (e.g. Cohen's *d*, Pearson's *r*), indicating how they were calculated

Our web collection on statistics for biologists contains articles on many of the points above.

Software and code

Policy information about availability of computer code

Data collection	Microscopy, IHC: Zen 2.3, Version 2.3.64.0, Zeiss GmbH<br>Immuno blots: ImageLab 6.1, Version 6.1.0 build 7, BioRad Laboratories<br>qRT-PCR: CFX Manager, Version 2.1.1022.0523, BioRad Laboratories
Data analysis	immuno blots and dotblots: ImageLab 6.1, Version 6.1.0 build 7, BioRad Laboratories<br>qRT-PCR: CFX Manager, Version 2.1.1022.0523, BioRad Laboratories<br>qRT-PCR, sorting of dataset: Excel 2016, Version 16.0.5378.1000, Microscoft<br>Graphs and statistics: Prism 7, Version 7.05, GraphPad Software, Inc.<br>Assembling of Schematic: BioRender, biorender.com<br>Assembling of figures: Photoshop 2020, Version 21.0.3<br>IHC length measurements & OHC cell count: ImageJ, Version 1.53f51, NIH

For manuscripts utilizing custom algorithms or software that are central to the research but not yet described in published literature, software must be made available to editors and reviewers. We strongly encourage code deposition in a community repository (e.g. GitHub). See the Nature Portfolio guidelines for submitting code & software for further information.

Data

Policy information about availability of data

All manuscripts must include a data availability statement. This statement should provide the following information, where applicable:

- Accession codes, unique identifiers, or web links for publicly available datasets
- A description of any restrictions on data availability
- For clinical datasets or third party data, please ensure that the statement adheres to our policy

All data generated or analyzed during this study are included in this article and in its supplementary information file. The source data for all graphs is provided as a compiled Excel sheet

Research involving human participants, their data, or biological material

Policy information about studies with human participants or human data. See also policy information about sex, gender (identity/presentation), and sexual orientation and race, ethnicity and racism.

Reporting on sex and gender	NA
Reporting on race, ethnicity, or other socially relevant groupings	NA
Population characteristics	NA
Recruitment	NA
Ethics oversight	NA

Note that full information on the approval of the study protocol must also be provided in the manuscript.

Field-specific reporting

Please select the one below that is the best fit for your research. If you are not sure, read the appropriate sections before making your selection.

☒ Life sciences ☐ Behavioural & social sciences ☐ Ecological, evolutionary & environmental sciences

For a reference copy of the document with all sections, see nature.com/documents/nr-reporting-summary-flat.pdf

Life sciences study design

All studies must disclose on these points even when the disclosure is negative.

Sample size	The sample size of each individual experiment are stated in the manuscript. For quantitative experiments, we did not perform sample size/ power analysis calculations prior to the start of the study. Sample sizes are typically larger for physiological and functional measurements (such as electroretinography) where past experience has shown there is more inter-animal variability. In past functional studies, sample sizes in the range of 5-10 animals per group have been sufficient to detect (with statistical significance) differences between means on the order of ~15-20%, so we use that as a starting target goal for each sample size. In practice, many sample groups for physiological studies are larger, this arises due to random differences in group size (when a cohort of animals reaches the right age after treatment we typically perform analyses on all animals available at that time point to avoid potential bias from selecting only certain animals). In this study and for its translatability potential, we used larger sample sizes. For structural studies (e.g. electron microscopy) we used a sample size of 2 due to the cost and difficulty of increasing the sample size.
Data exclusions	NONE was excluded
Replication	At least three replicates were made. For quantitative experiments a bigger sample size was selected when possible. Sample sizes are stated in submitted study for each experiment. The observation stated in the study were reproducible.
Randomization	Study animals were divided into groups, untreated, treated with saline, treated with control ASO and group treated with targeting ASO. Comparisons were made between the different groups and presented as such in the manuscript.
Blinding	For all functional and structural analyses, animals were only identified by tag number, not genotype or age.

Reporting for specific materials, systems and methods

We require information from authors about some types of materials, experimental systems and methods used in many studies. Here, indicate whether each material, system or method listed is relevant to your study. If you are not sure if a list item applies to your research, read the appropriate section before selecting a response.

Materials & experimental systems

n/a	Involved in the study
[ ]	[x] Antibodies
[x]	[ ] Eukaryotic cell lines
[x]	[ ] Palaeontology and archaeology
[ ]	[x] Animals and other organisms
[x]	[ ] Clinical data
[x]	[ ] Dual use research of concern
[x]	[ ] Plants

Methods

n/a	Involved in the study
[x]	[ ] ChIP-seq
[x]	[ ] Flow cytometry
[x]	[ ] MRI-based neuroimaging

Antibodies

Antibodies used	Sources and any other information regarding primary antibodies (Anti-rhodopsin, anti-perpherin 2, anti-GFAP) are provided in the table in the manuscript. Secondary Antibodies:<br>Alexa Fluor-555, donkey-anti-rabbit, Catalog number: A32794, Lot number: TI271031, Supplier: Invitrogen<br>Alexa Fluor-594, donkey-anti-rat, Catalog number: A21209, Lot number: 1547508, Supplier: Life Technologies<br>Goat Anti-Rabbit IgG Antibody, HRP conjugate, Catalog number: AP187P, Lot number: 2709676, Supplier: Sigma-Aldrich<br>Primary Antibodies:<br>Provided in supplementary table 1
Validation	Anti-Rhodopsin: Sigma Aldrich. (https://www.sigmaaldrich.com/US/en/product/sigma/r5403?utm_source=google&utm_medium=cpc&utm_campaign=8939553839&utm_content=95036811829&gclid=EAIaIQobChMIvN6H_tyhQMVSiWtBh1BrgonEAAYAiAAEgInBfD_BwE). Clone 1D4. Citations are at (https://www.sigmaaldrich.com/US/en/product/sigma/r5403?utm_source=google&utm_medium=cpc&utm_campaign=8939553839&utm_content=95036811829&gclid=EAIaIQobChMIvN6H_tyhQMVSiWtBh1BrgonEAAYAiAAEgInBfD_BwE#product-documentation). Validated in knockout mice.<br>anti-prphe2<br>Anti-GFAP. GTX636725. From GeneTex (https://www.genetex.com/Product/Detail/GFAP-antibody-HL1307/GTX636725). Various tissue extracts (50 µg) were separated by 10% SDS-PAGE, and the membranes were blotted with GFAP antibody [HL1307] (GTX636725) diluted at 1:3000. Also, tested in cells transfected with a GFAP construct and tested on immunoblots with extracts from untransfected cells as controls.

Animals and other research organisms

Policy information about studies involving animals; ARRIVE guidelines recommended for reporting animal research, and Sex and Gender in Research

Laboratory animals	Mice. C57BL/6
Wild animals	No wild animals were used
Reporting on sex	Both sexes were used and no sex differences were observed
Field-collected samples	No field animals were collected or used
Ethics oversight	All animal experiments were approved by the University of Houston Institutional Animal Care and Use Committee (IACUC) and adhered to NIH guidelines and the recommendations from the Association for Research in Vision and Ophthalmology (Rockville, MD).

Note that full information on the approval of the study protocol must also be provided in the manuscript.

Plants

Seed stocks	NA
Novel plant genotypes	NA
Authentication	NA
